# Carbamoylated Erythropoietin-Induced Cerebral Blood Perfusion and Vascular Gene Regulation

**DOI:** 10.3390/ijms241411507

**Published:** 2023-07-15

**Authors:** Jayanarayanan Sadanandan, Monica Sathyanesan, Yutong Liu, Neeraj K. Tiwari, Samuel S. Newton

**Affiliations:** 1 Division of Basic Biomedical Sciences, Sanford School of Medicine, University of South Dakota, Vermillion, SD 57069, USA; jayan.sadanandan@usd.edu (J.S.); monica.sathyanesan@usd.edu (M.S.); 2 Radiology Research Division, Department of Radiology, Nebraska Medical Center, Omaha, NE 68198, USA; yutongliu@unmc.edu; 3 The Jackson Laboratory, Sacramento, CA 95838, USA; neeraj.tiwari@jax.org

**Keywords:** CEPO, cerebral blood perfusion, MRI, vasodilation

## Abstract

Cerebral hypoperfusion is associated with enhanced cognitive decline and increased risk of neuropsychiatric disorders. Erythropoietin (EPO) is a neurotrophic factor known to improve cognitive function in preclinical and clinical studies of neurodegenerative and psychiatric disorders. However, the clinical application of EPO is limited due to its erythropoietic activity that can adversely elevate hematocrit in non-anemic populations. Carbamoylated erythropoietin (CEPO), a chemically engineered non-erythropoietic derivative of EPO, does not alter hematocrit and maintains neurotrophic and behavioral effects comparable to EPO. Our study aimed to investigate the role of CEPO in cerebral hemodynamics. Magnetic resonance imaging (MRI) analysis indicated increased blood perfusion in the hippocampal and striatal region without altering tight junction integrity. In vitro and in vivo analyses indicated that hippocampal neurotransmission was unaltered and increased cerebral perfusion was likely due to EDRF, CGRP, and NOS-mediated vasodilation. In vitro analysis using human umbilical vein endothelial cells (HUVEC) and hippocampal vascular gene expression analysis showed CEPO to be a non-angiogenic agent which regulates the MEOX2 gene expression. The results from our study demonstrate a novel role of CEPO in modulating cerebral vasodilation and blood perfusion.

## 1. Introduction

Erythropoietin (EPO), widely studied for its erythropoietic effects, has been shown to exert robust neuroprotective and neurotrophic effects in the CNS [1,2]. These neuronal effects are implicated in the behavioral response of EPO, including antidepressant [3] and cognitive effects [4]. The behavioral effects of EPO have been tested in both preclinical and clinical CNS studies. A double-blinded randomized clinical trial in a treatment-resistant depression population using EPO reported an improvement in depression scores and cognition [1]. Another study by the same group reported that EPO improved memory, attention, and executive functions after eight weeks of treatment for bipolar disease [2]. In addition to its neurotrophic effects, EPO has been reported as an angiogenic factor that enhances neovascularization in the ischemic brain [5]. The CNS actions of EPO are likely mediated by signaling via its cognate receptor: the erythropoietin receptor (EPOR). In addition to neurons, EPOR is also expressed in endothelial cells and is involved in stabilizing endothelial structures and vascular integrity [6,7].

Despite the promising results from EPO studies demonstrating its role in improving behavioral performance, it is important to note that chronic administration to non-anemic patients can significantly raise red blood cell counts, increase blood viscosity, and lead to adverse cardiovascular and cerebrovascular consequences [8]. An important line of research in the field is therefore focused on identifying and developing derivatives that can dissociate the erythropoietic properties of EPO from its behavioral effects. Carbamoylated erythropoietin (CEPO) is a non-erythropoietic derivative of EPO that does not produce erythropoietic effects but maintains neurotrophic and behavioral effects comparable to EPO. CEPO’s ability to retain neurotrophic effects without impacting erythropoiesis is thought to occur through the activation of an EPOR-CD131 heteromer rather than the EPOR dimer [8,9]. Previous studies from our laboratory showed that the CEPO differentially regulates the gene expression in the dorsal and ventral hippocampus and improves cognition in the rodent social defeat stress model [10]. As with EPO, the behavioral effects of CEPO are interpreted as a function of neuronal activity.

Whether CEPO influences cerebrovascular perfusion in addition to its neuronal actions is currently not known. Cerebral vasculature and blood perfusion as a function of neurotrophic factor administration is under-studied in psychiatric neuroscience. The brain is highly vascularized, and an adequate and well-regulated cerebral blood flow (CBF) is essential for normal brain function and could be compromised in neuropsychiatric disorders. Abnormal cerebral blood perfusion is reported to be associated with Alzheimer’s disease and major depressive disorders [11,12,13]. As a first step towards understanding the potential neurovascular effects of CEPO, we focused our attention on cerebral blood flow. We utilized magnetic resonance imaging (MRI) in mice to measure CEPO-induced changes in the brain. We complemented the imaging studies with in vivo gene expression in hippocampal blood vessels using laser microdissection. As cerebral blood vessels are composed of multiple cell types, we used human umbilical vein endothelial cells (HUVEC) to obtain mechanistic insight into CEPO’s vascular effects with resolution at the level of endothelial cells.

## 2. Results

### 2.1. Chronic CEPO Administration Increased Hippocampal and Striatal Blood Perfusion without Affecting BBB Integrity

**Figure 1:**
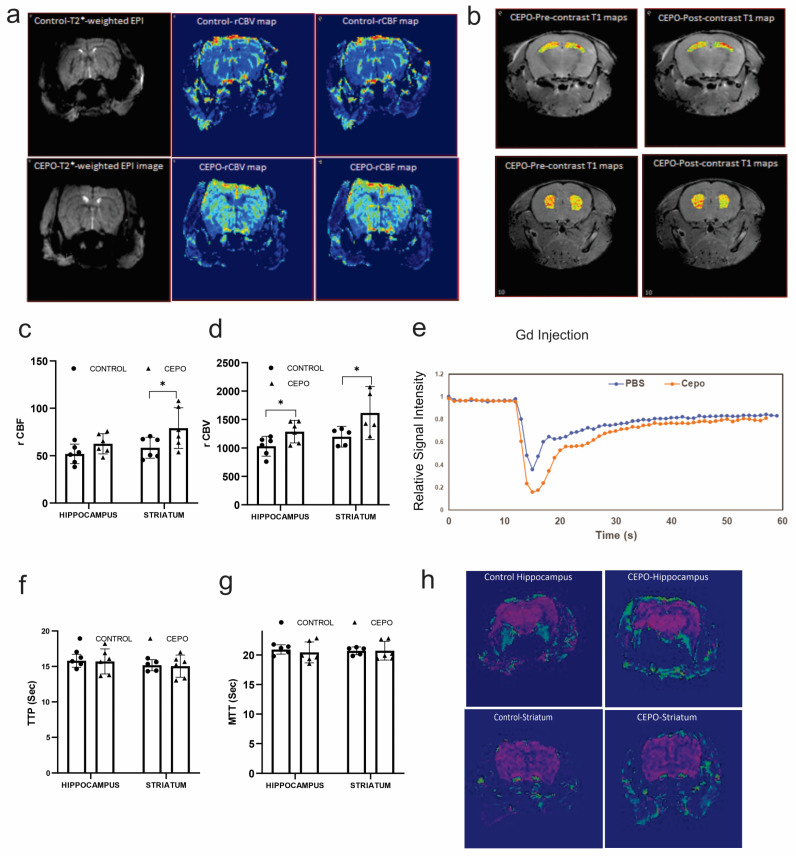
MRI mapping of hippocampal and striatum blood flow in chronic CEPO-treated mice: Perfusion parameter maps of chronic CEPO-treated mice (**a**). The heat maps of T1 in the hippocampus and striatum are superimposed on an anatomical image (**b**). Pre- and post-contrast T1 values in both regions. The ROI analysis of T2*-weighted EPI images showed a significantly increased rCBF in the striatum and an unchanged rCBF in the hippocampus of CEPO mice (**c**). rCBV (**d**) is significantly increased in the hippocampal and striatum of CEPO-treated mice. Gadolinium injection caused a signal drop in both chronic CEPO and PBS-treated mice (**e**). Time to peak (TTP) (**f**) and Mean transit time (**g**) was unchanged in the CEPO-treated mice. K2 leakage map (**h**) of CEPO-treated mice showed no leakage, indicating intact BBB. Data are mean ± SD, *n* = 6 animals/group. Adjusted *p*-values are indicated as * *p* ≤ 0.05.

The effect of CEPO administration on cerebral circulation was examined using an MRI. Increased blood perfusion was observed in mice with chronic CEPO administration compared to the controls. The ROI analysis of T2*-weighted EPI images showed (Figure 1a) a significant increase in the hippocampal (*p* = 0.023) and striatal (*p* = 0.012) relative cerebral blood volume (rCBV) of CEPO-treated mice (Figure 1d). Regional cerebral blood flow (rCBF) showed a trend of increase in the hippocampus (*p* = 0.055) and a significant increase in the striatum (*p* = 0.044) of CEPO mice (Figure 1c). Gadolinium injection caused a signal drop in both chronic CEPO and PBS-treated mice. The signal drop was deeper and wider in CEPO-treated mice compared to PBS-treated controls (Figure 1e). There was no significant difference in mean transit time (MTT) (Figure 1g) and time to peak (TTP) (Figure 1f) between the two groups. The heatmaps of T1 on the hippocampus and striatum were superimposed on an anatomical image (Figure 1b). The ROI analysis found no significant difference between pre- and post-contrast T1 on the hippocampus (pre-contrast T1 = 2103 ± 84 ms vs. post-contrast T1 = 2010 ± 79 ms) and striatum (pre-contrast T1 = 1790 ± 69 ms vs. post-contrast T1 = 1814 ± 41 ms). No significant difference was found between pre- and post-contrast T1 values in both CEPO-treated mice and the control. Consistent pre- and post-contrast T1 values, along with no gadolinium leakage in the brain vasculature from the K2 map (Figure 1h) confirmed intact BBB in CEPO-treated mice. Acute administration of CEPO did not show any significant change in the brain blood perfusion (Supplementary Figure S1).

### 2.2. CEPO Elevated HIF1A Gene Expression without Elevating Angiogenic Factors in HUVECs

**Figure 2:**
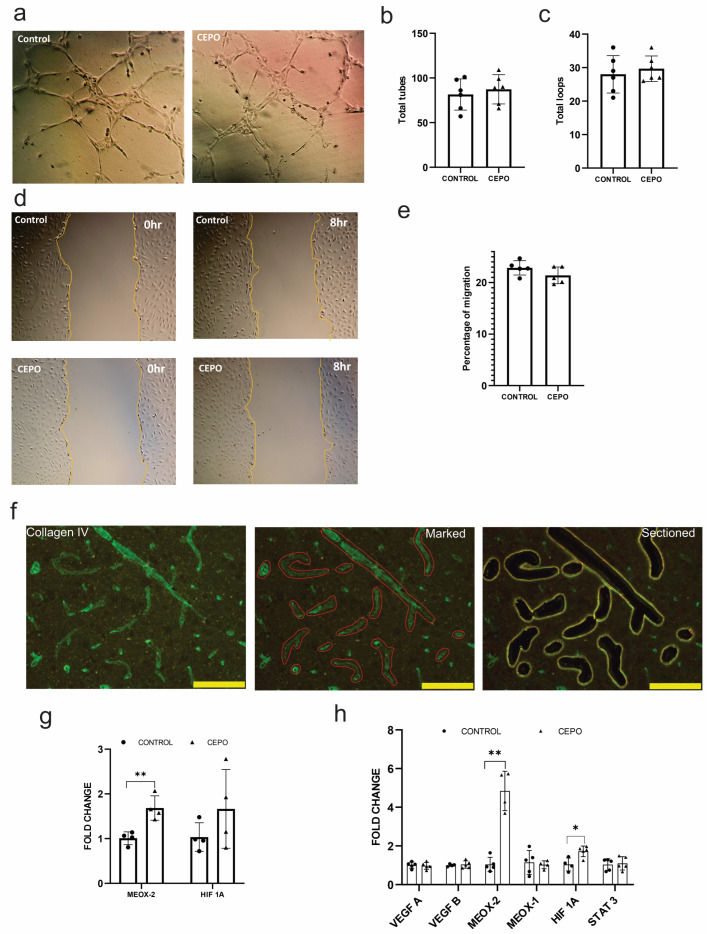
In vitro and in vivo analysis of Angiogenic properties of CEPO: representative images for tube formation assay (**a**). The total number of tubes (**b**) and loops (**c**) formed during CEPO treatment is measured using ImageJ 1.53t. Representative images for scratch assay (**d**). Representative images for Migration assay 0 h and 8 h (**e**). Both the control and CEPO-treated HUVECs showed 18–20% migration. Representative images of blood vessels (Green) stained with vascular marker collagen IV Scale bar = 100 μm (**f**). Blood vessels were marked and sectioned using LMD followed by RNA isolation and gene expression analysis of pro-angiogenic factor MEOX2 and HIF-1 in the blood vessels of the control and CEPO-treated rats (*n* = 6/group) (**g**). Expression of Pro-angiogenic genes in the control and CEPO-treated HUVECs (*n* = 4–5/group) (**h**). Data are mean ± SD. The adjusted *p*-value is indicated as * *p* ≤ 0.05, ** *p* ≤ 0.01.

To examine whether increased hippocampal and striatal blood flow is due to angiogenesis, we investigated the angiogenetic activity of CEPO. To study angiogenesis at the molecular level, we examined the gene expression of angiogenic factors. Hypoxia is one of the important factors that promote vessel growth by activating multiple pro-angiogenic pathways. We examined the gene expression of Hypoxia-inducible factor 1-alpha (HIF 1α), an oxygen-dependent transcriptional activator that plays a vital role in angiogenesis. LMD-sectioned rat blood vessels (Figure 2f) showed a tendency of increased HIF 1α gene expression but were not statistically significant (Figure 2g). HIF 1a expression was significantly (*p <* 0.01) upregulated (Figure 2h) in CEPO-treated HUVECs, indicating a favorable condition for angiogenesis. Angiogenic factors vascular endothelial growth factor A (VEGF A) and VEGF B, along with the VEGF transcription activator signal transducer and activator of transcription 3 (STAT3) expression patterns, remain unaltered (Figure 2h) in the CEPO-treated HUVECs. Furthermore, we examined the expression of mesenchyme homeobox 1 (MEOX1) and MEOX2, a family of diverged homeodomain transcription factor proteins, which are robustly expressed in endothelial cells and negatively regulate vascular cell proliferation. MEOX2 gene expression) was significantly (*p* < 0.0001) upregulated (Figure 2h) in the CEPO-treated HUVECs, while MEOX1 expression remained unchanged (Figure 2h). Blood vessels of CEPO-treated rats also showed a significantly (*p* < 0.004) elevated MEOX2 gene expression (Figure 2g). To further confirm the role of CEPO in angiogenesis, a tube formation assay (Figure 2a) and wound healing assay (Figure 2e) were performed in CEPO-treated and nontreated HUVECs. The tube formation ability of the HUVECs was not affected by CEPO treatment. There was no significant alteration (Figure 2b,c) in the number of tubes and loops between CEPO-treated and non-treated HUVECs, indicating the non-angiogenic effect of CEPO. Migration of the endothelial cells (Figure 2d) showed a similar pattern of results: both control and CEPO treatment in HUVECs showed 19–20% migration (Figure 2e) after 8 h of incubation.

### 2.3. CEPO Differentially Regulates the Transcription of Neurotrophic Factors in the HUVECs

**Figure 3:**
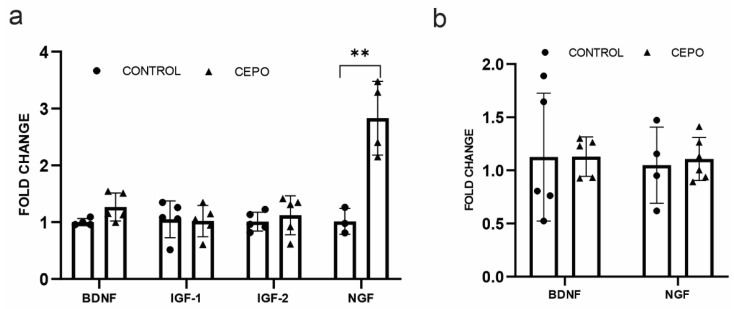
Neurotrophic factors gene expression: mRNA expression of neurotrophic factors BDNF, IGF-1, IGF-2, and NGF in the control and CEPO-treated HUVECs (*n* = 4–5/group) (**a**). Gene expression of the neurotrophic factors BDNF and NGF in the LMD sectioned control and CEPO-treated rat blood vessels (*n* = 6/group) (**b**). Gene expression was normalized using the housekeeping gene GAPDH and Beta-actin for HUVEC and LMD sections, respectively. Data are mean ± SD. The adjusted *p*-value is indicated as ** *p* ≤ 0.01.

Endothelial cells produce trophic factors which are involved in angiogenesis and vasodilation. Blood vessels of CEPO-treated rats did not show any alterations in the neurotrophic factors, the brain-derived neurotrophic factor (BDNF), or the nerve growth factor (NGF) gene expression (Figure 3b). However, NGF (Figure 3a) mRNA transcription was significantly upregulated (*p* < 0.006) in CEPO-treated HUVECs. The expression of BDNF and IGF (Insulin-like growth factor) 1 and 2 (Figure 3a) did not show any significant change in CEPO-treated HUVECs. 

### 2.4. CD131 and EPO Receptor Gene Expression Is Elevated in CEPO-Treated HUVECs and Unchanged in the Dentate Gyrus

**Figure 4:**
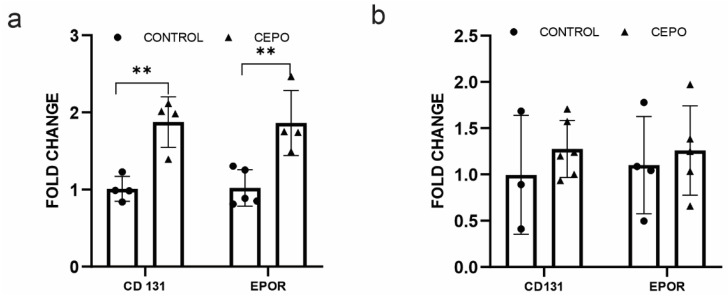
EPOR and CD131 gene expression: CD131 and EPOR gene expression in the control and CEPO-treated HUVECs (*n* = 4–5/group): (**a**). CD131 and EPOR gene expression in the LMD sections of control and CEPO-treated rat blood vessels (*n* = 6/group) (**b**). Gene expression was normalized using the housekeeping gene GAPDH and Beta-actin for HUVEC and LMD sections, respectively. Data are mean ± SD. The adjusted *p*-value is indicated as ** *p* ≤ 0.01.

CEPO has been proposed to bind an EPOR homodimer or EPOR-βc receptor (*CD131*) heteromer complex to activate the molecular response. To examine the changes in EPOR and cd131 receptors during CEPO administration, gene expression analysis of EPOR and CD131 was carried out in the HUVECs and BV of CEPO-administrated rats. EPOR (*p* < 0.006) and CD131 (*p* < 0.003) gene transcription was significantly elevated in the CEPO-treated HUVECs (Figure 4a), but unchanged in the BV (Figure 4b).

### 2.5. Glutamatergic and Dopaminergic Receptor Expression Was Intact in CEPO-Treated Rats

**Figure 5:**
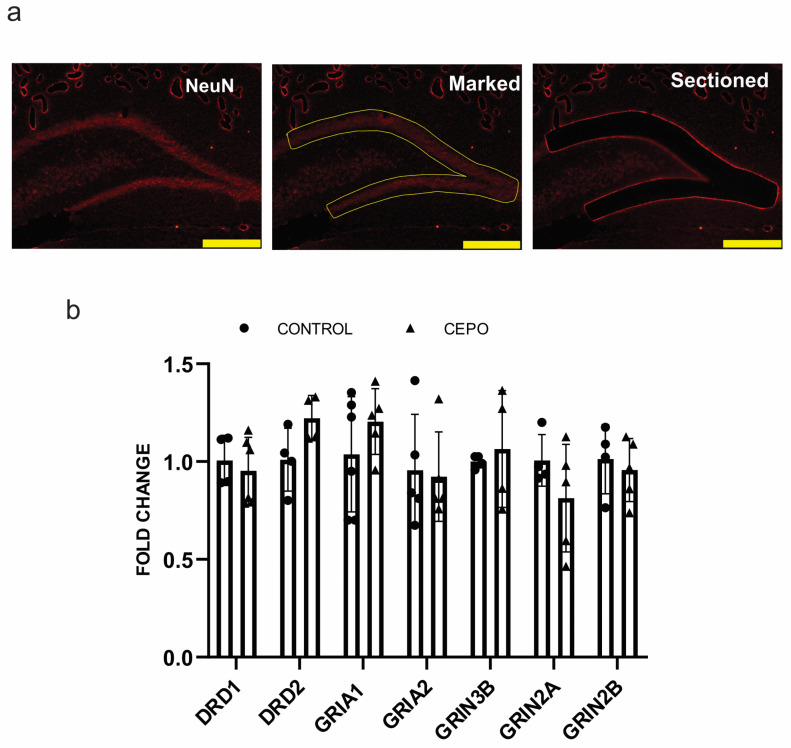
Neurotransmitter receptor gene expression in the dentate gyrus (DG) of CEPO-treated mice. Representative images for immunofluorescence laser micro-dissection (IF-LMD) of the dentate gyrus Scale bar = 400 μm (**a**). Neurons stained with NeuN (red) primary antibody followed by Alexa flour 594. Neurotransmitter receptor gene expression in the DG of the control and CEPO-treated rat DG (**b**). Gene expression is normalized using the housekeeping gene Beta-actin Data are mean ± SD, *n* = 6/group.

Gene expression analysis of glutamatergic and dopaminergic gene expression was carried out in the dentate gyrus to identify whether the increased blood perfusion was due to the hyperactivation of the ionotropic glutamate and dopamine receptors. Rat rain is stained with NeuN (Figure 5a) and DG is dissected out using LMD. Dopaminergic receptor D1 expression was unaltered in the CEPO-treated rat DG (Figure 5b). Dopamine receptor D2 showed a trend towards upregulation but was not statistically significant (*p* < 0.07) (Figure 5b). The α-amino-3-hydroxy-5-methyl-4-isoxazole propionic acid (AMPA) receptor subunit receptor Glutamate Ionotropic Receptor AMPA Type Subunits 1 and 2 (GRIA1 and GRIA2) gene expression were unchanged in the dentate gyrus (DG) of CEPO-treated rats (Figure 5b). CEPO treatment did not produce any alterations in the N-methyl-D-aspartic acid (NMDA)receptor subunits GRIN2A, GRIN2B, and GRIN3A mRNA transcription (Figure 5b).

### 2.6. CEPO Promotes Vasodilation by Transcriptionally Regulating EDRF and CGRP Genes

**Figure 6:**
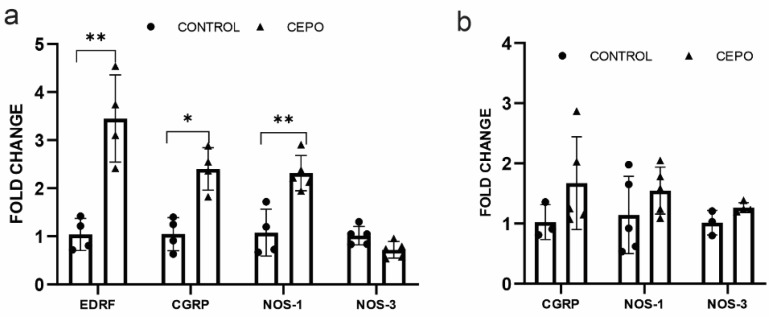
Vasodilation factors mRNA expression in the control and CEPO-treated HUVECs (*n* = 4–5/group) (**a**). Vasodilation factor mRNA expression in the blood vessels of control and CEPO-treated rat blood vessels (*n* = 6/group) (**b**). Gene expression was normalized using the housekeeping gene GAPDH and Beta-actin for HUVEC and LMD sections, respectively. Data are mean ± SD. The adjusted *p*-value is indicated as * *p* ≤ 0.05, ** *p* ≤ 0.01.

To identify whether the increased CBF perfusion during CEPO administration is due to vasodilation, we performed gene expression analysis of the following established vasodilators genes such as endothelium-derived relaxing factor (EDRF), calcitonin gene-related peptide (CGRP), and nitric oxide synthase 1 and 3 (NOS1, and NOS3). Vasodilation factor CGRP expression showed a trend of increase in the CEPO-administrated rat blood vessels (Figure 6b). In vitro, our study showed a significantly increased CGRP (*p* < 0.002) and EDRF (*p* < 0.002) mRNA transcription in CEPO-treated HUVECs (Figure 6a). A common endothelial vasodilator NOS3 expression showed an increased trend (*p* < 0.07) in the blood vessels of CEPO-administrated rats (Figure 6b), but NOS3 expression was unchanged in the vascular endothelial cells (Figure 6a). Neuronal NOS (NOS-1) is expressed in the vascular endothelial cells. CEPO treatment significantly elevated (*p* < 0.001) NOS-1 gene expression (Figure 6a) in HUVECs while in blood vessels it remained constant (Figure 6b).

## 3. Discussion

A healthy blood flow is vital for optimal brain function. Stable and well-regulated blood flow brings oxygen, glucose, and necessary nutrients into the brain and carries carbon dioxide and other metabolic by-products away from the brain. Neurotrophic factors are well known to promote the growth, survival, and function of neurons [14,15,16,17]. However, their actions in non-neuronal cell types, particularly cerebrovasculature, have not received much attention. The behavioral actions of EPO have gained significant momentum due to several preclinical and clinical studies reporting antidepressant-like and neurocognitive effects. Despite its remarkable safety profile and wide use in the treatment of anemia, EPO’s potent erythropoietic properties limit its development as a CNS drug. The discovery of non-erythropoietic CEPO was significant as it provided the opportunity to safely augment trophic activity in the brain. Recent rodent behavioral experiments showing that CEPO reproduces the behavioral effects of EPO imply that this is likely due to their overlapping neurotrophic profile and neuronal mechanisms [18]. In this study, we focused our attention on the neurovascular properties of CEPO, particularly its role in cerebral blood perfusion. MRI analysis showed an increase in cerebral blood flow in the hippocampus and striatum of chronic CEPO-treated mice without affecting BBB integrity. Acute treatment of CEPO did not show any significant change, indicating that a single dose of CEPO is not sufficient to impact hemodynamic changes in the brain. We sought to gain further insight into CEPO’s vascular actions due to elevated CBV and CBF. We hypothesized that increased blood perfusion from chronic CEPO administration could be due to either angiogenesis, vasodilation, or the hyperactivation of neurotransmitter receptors.

Carbamoylated EPO is thought to signal via an EPOR-CD131 heterodimer rather than the EPOR-EPOR homodimer [19,20,21,22]. We examined the mRNA expression levels of EPOR and CD131 in the HUVECs and blood vessels. Both the EPOR and CD 131 receptor’s expression were unaltered in the BV of the CEPO-treated rats, while EPOR and CD131 gene expression showed a significant upregulation in the vascular endothelial cells. This could be due to a temporal pattern of gene regulation in BV that is related to the kinetics of CEPO entry into the brain after systemic administration and the challenge of capturing endothelial cell-specific gene expression changes in vivo. 

We first investigated the possibility of angiogenic mechanisms to determine whether the increased blood perfusion is due to the formation of new blood vessels. Interestingly, blood vessels of CEPO-administrated rats showed an increase in HIF 1α mRNA transcription. CEPO-treated HUVECs also showed a significantly elevated HIF 1α gene expression, indicating the possibility of a hypoxia-independent condition favorable to angiogenesis [23,24]. HIF 1α can also enhance endothelial migration, growth, and differentiation [25]. We examined angiogenesis-related gene transcription in the endothelial cells to determine whether elevated HIF 1α expression triggers the activation of pro-angiogenic genes. Normally, under hypoxic conditions, VEGF—a key mediator of angiogenesis—is expressed robustly in the endothelial cells and promotes angiogenesis [25,26]. However, VEGF A and VEGF B expressions remained unchanged in the CEPO-treated endothelial cells. The consistent expression of VEGF in CEPO-treated endothelial cells could be due to an inactive signal transducer and activator of transcription 3 (STAT3). STAT3 plays a crucial role in angiogenesis by regulating VEGF expression [27,28,29,30]. Previous reports indicate that the EPOR homodimer activates STAT3 in neurons and glia [31,32]. As a non-erythropoietic EPO derivative, CEPO could act through an EPOR-CD131 heterodimer-driven cascade [9] that does not cause STAT3 activation. However, conclusive evidence requires a focused analysis of STAT3 phosphorylation status in neurons and vasculature.

Homeobox genes are master regulatory genes that have diverse functions in different cell types during the embryonic, as well as the adult, stage [33,34,35,36]. The mesenchyme homeobox genes, MEOX1 and MEOX2 are expressed both in vascular endothelial cells and smooth muscle cells with properties, indicating that it is a master regulatory gene controlling the angiogenic phenotype. LMD gene expression analysis showed elevated MEOX2 expression in the CEPO-treated rat blood vessels. A similar pattern of results was observed in the CEPO-treated HUVECs. MEOX2 expression is significantly low in Alzheimer’s disease neuro vasculature, causing vessel malformation and regression leading to reduced capillary density and CBF [37]. It would be worthwhile to investigate CEPO in AD to test whether it can reverse MEOX2 downregulation and rescue dysregulated vasculature. The absence of a CEPO-induced effect in the migration and tube formation assays enabled us to confirm that CEPO is likely non-angiogenic. The collective evidence from our study indicates that CEPO increases cerebrovascular perfusion without impacting angiogenic mechanisms. 

We tested the possibility that increased neuronal activity in the hippocampus modulates elevated blood flow. In the brain, neuronal activation is accompanied by an increase in perfusion. The augmented neural activity in the brain is typically coupled with surges in cerebral blood flow leading to the increased oxygenation that generates a bold MRI signal. Here we particularly focused on the ionotropic glutamatergic and dopamine receptor transcription in the hippocampal dentate gyrus (DG). We analyzed the transcription of glutamatergic AMPA and NMDA receptor subunits along with Dopamine receptors D1 and D2 in CEPO-treated brains. Unchanged subunit-level expression of neurotransmitter receptors in DG of the CEPO-treated animals suggested that neurotransmitter hyperactivation is likely not involved in the CBF increase. 

We then examined the involvement of vasodilation in CEPO-induced increase in CBF. The vascular endothelial layer of micro vessels is considered a key regulator of vasodilation through the synthesis and release of vasoactive substances [38,39]. LMD analysis sheds light on CEPO-induced gene expression changes of potential vasodilators in the brain blood vessels whereas HUVECs provide resolution at the level of endothelial cells We feel this combination approach provides useful insight into CEPO’s vascular actions. Vasodilator EDRF gene expression was elevated in CEPO-treated HUVECs. Vascular endothelial cells produce EDRF, which promotes vasodilation and inhibits platelet aggregation and adherence to the vascular endothelial surface [40]. The elevated EDRF causes the relaxation of vascular smooth muscle cells through the production of cyclic guanosine monophosphate (cGMP) followed by the activation of protein kinase [41]. The induction of HIF-1a could potentially drive EDRF expression in CEPO-treated HUVCS. Vascular endothelial cells produce EDRF in response to stress signals such as hypoxia and adenosine 5’-diphosphate (ADP) accumulation [42]. Another vasodilation molecule CGRP expression increased in the blood vessels of CEPO-treated animals but was not statistically significant. However, it was significantly regulated in HUVECs. Endothelial cells produce and store the neuropeptide CGRP in Weibel–Palade bodies, which are also involved in autoregulation and hemodynamics [43,44]. CGRP mainly exerts its vasodilatory effects through a nitric oxide-dependent vasodilator mechanism [45]. Some reports also suggest that the CGRP receptor diminishes the effects of the potent constrictor agent endothelin via an interaction involving the G-protein βγ subunit [46]. We propose that CEPO-enhanced EDRF and CGRP expression in the endothelial cells promotes the local relaxation of vascular smooth muscles through NOS-1-mediated NO production resulting in increased cerebral blood flow.

Several reports suggest that cerebral hypoperfusion-associated cognitive impairment is a common phenomenon in neuropsychiatric disorders such as dementia, schizophrenia, and AD [47,48,49]. Our study demonstrates that CEPO increases blood flow in the hippocampus and striatum without altering tight junction integrity and hippocampal neurotransmission. CEPO increased the expression of pro-vasodilator genes in endothelial cells and likely plays an integral role in the production of vasodilators. A limitation of our study is that the assessment of hippocampal neuronal activation was limited to changes in gene regulation. Both in vitro and in vivo data point to EDRF and CGRP and NOS-1-mediated vasodilation as the potential mechanism in CEPO-induced perfusion. We have previously shown that CEPO produces cognitive [50] and antidepressant-like effects [51]. Given the importance of cerebrovascular parameters to brain function, future studies should aim to determine the contribution of CEPO-induced cerebral blood flow to the behavioral response. Considering the potential of CEPO as a vasodilating agent that maintains BBB integrity and lacks erythropoietic as well as angiogenic activity, it can be a useful target molecule for testing in conditions of cerebrovascular perfusion deficits.

## 4. Materials and Methods

### 4.1. Carbamoylation of EPO

Erythropoietin was purchased from Prospec Bio (Ness-Ziona, Israel) and carbamoylated in 1 mg aliquots as previously reported [18,50]. Briefly, EPO was deprotonated in a high pH (pH = 8.9) borate buffer and then exposed to potassium cyanate for 16 h at 36 °C. CEPO was exhaustively dialyzed for 6 h against PBS. CEPO concentration was determined using the Qubit protein assay (Thermo Fisher, Waltham, MA, USA). CEPO purity was verified by silver staining after electrophoretic gel analysis.

### 4.2. Cell Culture

Human umbilical vein endothelial cells (HUVEC cells) were obtained from the American Type Culture Collection (ATCC, Manassas, VA, USA). The cells were grown in DMEM/F12 (ATTC, Elizabeth City, NC, USA) supplemented with ECGS (0.05 mg/mL) (Corning, Corning, NY USA) and heparin (0.1 mg/mL) (Sigma, St. Louis, MO, USA) with 20% FBS at 37 °C and 5% CO_2_ on a collagen type 1 (Sigma)-coated cell culture dish. HUVECs were treated with CEPO 100 ng/mL for 3 h. Vehicle-treated (PBS) HUVECs were used as controls. Three to five replicates were used for each control and CEPO-treated sample for the gene expression studies.

### 4.3. RNA Extraction and Quantitative PCR Analyses in HUVEC

Total RNA from the CEPO-treated and nontreated HUVECs were extracted using Trizol^®^ RNA isolation reagent (Invitrogen, Waltham, MA, USA) according to the manufacturer’s instructions. NanoDrop ND-1000 spectrophotometer (Thermo Scientific, Wilmington, DE, USA) was used to determine the concentration and purity of RNA at 260/280 nm. For the quantitative gene expression analyses, 500 ng RNA was reverse transcribed into cDNA (Applied Biosystems High-Capacity cDNA Reverse Transcription Kit, Foster City, CA, USA) using thermal cyclers (Techne Cole-Parmer, Vernon Hills, IL, USA). Gene expression analyses were performed by quantitative real-time PCR (applied biosystems QuantStudio 5 and Eppendorf Mastercycler Realplex 2) using 500 nM of each forward and reverse primers (Supplementary Table S1) and SYBR green Universal PCR master mix (Gendepot, Baker, TX, USA and Applied Biosystems, Waltham, MA, USA). Amplification conditions are 40× cycles of 94.0 °C for 2 s, 60.0 °C for 30 s, and 72.0 °C for 30 s. Primers for the amplification of gene targets were designed using the Primer3 program (https://bioinfo.ut.ee/primer3-0.4.0/, accessed on 20 October 2022). The expression level of each gene target was normalized with the housekeeping gene (Glyceraldehyde 3-phosphate dehydrogenase (GAPDH)), and the fold change of transcription was quantified using the relative quantification 2^−∆∆Ct^ method.

### 4.4. Animals

Adult male Sprague Dawley rats (12 weeks old, *n* = 6 per group, mass 220–240 gm; Envigo, Indianapolis, IN, USA) were randomly paired and housed according to treatment group (Vehicle, and CEPO) for the duration of the experiments. We used the rat model for the laser microdissection (LMD) study to obtain a higher RNA yield. Rats were maintained on a standard 12 h light–dark cycle with free access to food and water. All procedures were carried out in strict accordance with the National Institutes of Health Guide for the care and use of laboratory animals and were approved by the USD Institutional animal care and use committee. Every effort was made to minimize the number of animals used. Rats received single daily i.p. injections of either vehicle (PBS), or CEPO (30 µg/kg) for 4 consecutive days [18,50]. Five hours after the last dose, animals were decapitated according to the American veterinary medical association guidelines. Brain samples were quickly frozen using dry ice and brain sections were collected on laser microdissection (LMD) slides (Leica pen membrane 2.0 µm) using Leica cryostat. LMD sections were used for laser microdissection and gene expression analysis. For the cerebral blood flow (CBF) MRI studies, we used 12-week-old male Balb/c mice. Both acute and chronic effects of CEPO were tested. The acute group (male Balb/c mice, *n* = 6) was treated with i.p. injections of 40 μg/kg CEPO and scanned after 3 h. The chronic group (*n* = 6) was injected with 5 doses of CEPO (i.p., 40 μg/kg/day), and scanned after 24 h of the last dose. Two control groups were treated with acute and chronic PBS injections, respectively. (Experimental design is provided in Supplementary Figure S2). The study was not blinded and had no animals excluded from the experiments. Reporting of these experiments complies with the ARRIVE (Animal Research: Reporting in Vivo Experiments) guidelines.

### 4.5. Laser Microdissection and RNA Preparation

Fresh frozen 16 µm hippocampal coronal brain sections were collected on LMD slides. Sections were fixed using a histochoice MB fixative (Electron microscopy, Berkeley, CA, USA). Blood vessels were stained using collagen IV primary antibody (Abcam AB6586, Cambridge, UK) for 45 min at room temperature followed by Alexa fluor 488 secondary antibodies (Thermo Fisher Scientific, Waltham, MA, USA) specific to primary for 30 min at room temperature. Neurons were stained using NeuN antibodies (Millipore MAB377, Burlington, MA, USA) followed by Alexa fluor 594 secondary antibodies (Thermo Fisher Scientific, USA) specific to primary. Stained blood vessels and DG were laser-micro dissected using the LMD7000 (Leica Microsystems, Wetzlar, Germany) and collected into the cap of a 0.5 mL (BIO-BIK BT-02LC, Ina-Optika, Osaka, Japan) microtube containing 50 µL lysis buffer. At every collection, the presence of the laser-micro dissected tissue sample in the cap of the microtube was confirmed by LMD microscopy. RNA was isolated using an RNAqueous micro kit (Invitrogen) according to the manufacturer’s protocol.

### 4.6. cDNA Preparation and Gene Expression

cDNA synthesis was carried out using the protocol of Pekas et al., 2020 [52]. Briefly, 1 µL Oligo DT20 Primer was added to the total RNA (volume of 12 µL) isolated from hippocampal and blood vessel LMD sections. The RNA- Oligo DT20 Primer mixture was heated at 80 °C for 10 min followed by 5 min incubation on ice. After the incubation, 4 µL 5× RT buffer, 1 µL 10 mM dNTP mix,1 µL SuperScript III Reverse Transcriptase, and 1 µL SUPERase Inhibitor were added to each sample. This was mixed well and incubated at 42 °C for two hours. The reaction was stopped by adding 0.5 M NaOH/50 mM EDTA followed by incubation at 65 °C for 10 min. After 10 min incubation 5 µL of 1 M Tris-HCL, 70.5 µL 10 mM Tris/1 mM EDTA, 3 µL of 5 mg/mL acrylamide, 4 µL 5 M NaCl, and 400 µL of 100% EtOH added, and the resulting mixture was incubated overnight at −20 °C. The sample was centrifuged at 10,000 rpm for 15 min to pellet out the cDNA. The pellet was washed using 450 µL of cold 70% EtOH and dried at 65 °C. RT-qPCR was performed using an Eppendorf Mastercycler RealPlex 2 (Hauppauge, New York, NY, USA) for 50× cycles of 94.0 °C for 2 s, 60.0 °C for 30 s, and 72.0 °C for 30 s. The primers used in the studies were designed using the Primer3 program. The expression level of each gene target was normalized with the housekeeping gene. Beta-actin Quantification was performed using SYBR Green chemistry. Only samples with appropriate melt curves were included in the study.

### 4.7. Brain Blood Flow Analysis Using Magnetic Resonance Imaging (MRI) and Data Analysis

The MRI was performed on a 7-tesla scanner (Bruker, Billeria, MA, USA). The measurements were conducted on animals under 2% isoflurane carried by 1 L/min of oxygen. The animals were placed on a thermal pad and their body temperatures and respiratory rates were monitored during the measurement process. The mice were first scanned with RARE VTR for pre-contrast T1 mapping. A dynamic susceptibility contrast (DSC) MRI was performed using a T2*-weighted single-shot EPI GRE with TR = 1 s and scan time = 60 s. Gd-DPTA was injected through the tail vein at 10 s with a dose = 0.1 mmol/kg. DSC was followed by post-contrast T1 mapping. Relative cerebral blood volume (rCBV), relative cerebral blood flow (rCBF), mean transit time (MTT), and time to peak (TPP) maps were generated using DSCoMAN [53] (https://sites.duke.edu/dblab/dscoman/, accessed on 24 May 2022). K2 maps were also generated. T1 maps were calculated using an in-house fitting program [54].

### 4.8. Tube Formation Assay

The Tube formation assay was carried out to determine the angiogenetic activity of CEPO. HUVECs were seeded onto 48-well plates (5 × 10^4^/well) previously coated with 75 μL of growth factor-reduced Geltrex (Thermo Fisher, USA), with or without 100 nm CEPO. The morphology of the capillary-like structures formed by the HUVECs was analyzed by an inverted microscope after 6 h of culture and digital images were captured. After 6 h of incubation, the total number of tubes and loops was quantified manually using imageJ 1.53t. 

### 4.9. Migration Assay

Each well of a collagen-coated 6-well plate (Corning) was seeded with 5 × 10^5^ HUVECs in DMEM/F12 supplemented with ECGS (0.05 mg/mL) and heparin (0.1 mg/mL) with 20% FBS. When the cells became confluent, a micro pipettor was used to generate a 1 mm wide scratch on the bottom of the 6-well. Cells were rinsed with PBS and incubated in DMEM (without growth supplement) with 100 ng/mL CEPO, and control groups were treated with PBS. Microscopy was used to observe and photograph cell migration during the 0 h and 8 h after CEPO supplementation. The scratch area was analyzed using ImageJ 1.53t to determine the percentage of migration.

### 4.10. Data Analysis

Statistical analysis was performed using GraphPad Prism 8.4 software. All statistical tests use biological replicates and are indicated by group size (*n*) in the figure legend. Results were expressed as mean ± SD (standard deviation). The following statistical tests were applied to determine statistical significance (*p* < 0.05): an unpaired two-tailed *t*-test was used for Q-PCR, a Tube formation, and a scratch assay analysis. The unequal variance was corrected using Welch’s correction (GraphPad Prism 8.4). An unpaired one-tailed *t*-test was used for MRI analysis.

## Data Availability

The data presented in this study are available on request from the corresponding author.

## Supplementary Materials

The supporting information can be downloaded at: https://www.mdpi.com/article/10.3390/ijms241411507/s1.
