# Supplementary material for: Carbamoylated Erythropoietin-Induced Cerebral Blood Perfusion and Vascular Gene Regulation
Source: Int J Mol Sci. 2023 Jul 15;24(14):11507. doi: 10.3390/ijms241411507 (PMC10380798; doi:10.3390/ijms241411507)
Supplement: Supplementary file 1 [file ijms-24-11507-s001.zip › supplimentary figure S-1.pdf]

MRI mapping of hippocampal and striatum blood flow in Acute CEPO treated mice

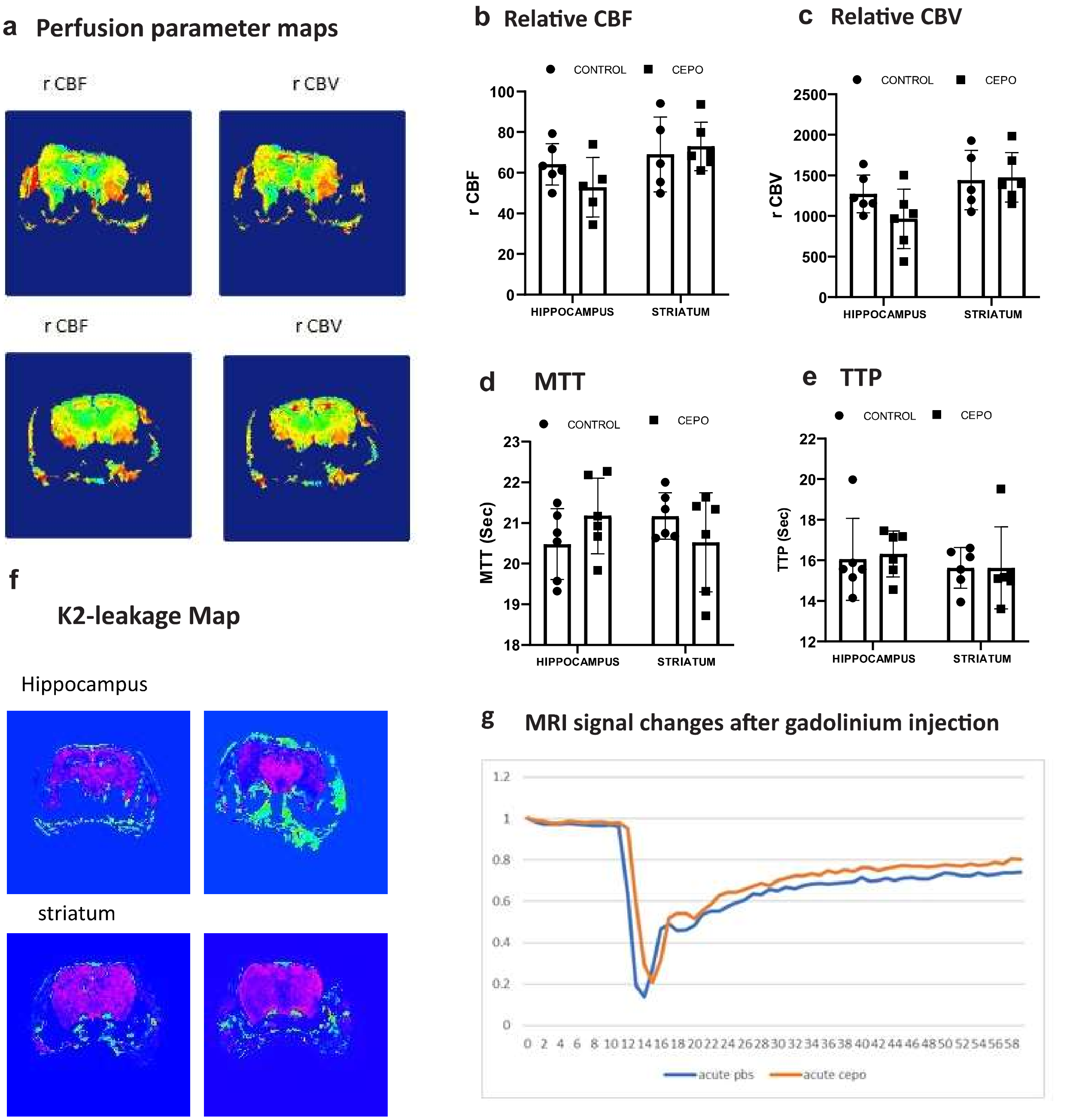

Figure S1 . MRI mapping of hippocampal and striatum blood flow in Acute CEPO treated mice : Perfusion parameter maps of chronic CEPO treated mice (a) Relative cerebral blood flow (b), and Relative cerebral blood volume (c) remains unchanged in the hippocampus and striatum of acute CEPO treated mice. Mean transit time (MTT) (d) and Time to peak (TTP) (e) was consistent in the Acute CEPO treated mice. K2 leakage map (f) of CEPO -treated mice showed no leakage indicating int act BBB. MRI signal changes after gadolinium injection in mice treated with chronic PBS and CEPO (g) signal drop was similar in the CEPO and PBS -treated mice indicating no change in blood perfusion .
