# Supplementary material for: Carbamoylated Erythropoietin-Induced Cerebral Blood Perfusion and Vascular Gene Regulation
Source: Int J Mol Sci. 2023 Jul 15;24(14):11507. doi: 10.3390/ijms241411507 (PMC10380798; doi:10.3390/ijms241411507)
Supplement: Supplementary file 1 [file ijms-24-11507-s001.zip › supplimentary table1.pdf]

Table S1. Primers used for qPCR

Gene symbol	Accession number	Primer sequence		Species
		Forward	Reverse	
STAT3	NM_139276.3	TCTGGGTCTGGCTGGACAAT	GCCTGGAGGCTTAGTGCTCA	Homo sapiens
HIF1a	NM_001530.4	AGTTCCGCAAGCCCTGAAAG	TGGTGGCAGTGGTAGTGGTG	Homo sapiens
GAPDH	NM_002046.7	CCACTCCTCCACCTTTGACG	CATGAGGTCCACCACCCTGT	Homo sapiens
MEOX2	NM_005924.5	CCCGTTCTCCCAATCCTCTC	CTGGCAAACATGCCCTCTTC	Homo sapiens
NOS1	NM_000620.5	GCCGGAGACATCATTCTTGC	GACCACGTGGGTCTCAGAGG	Homo sapiens
NOS3 TV3	NM_001160110.1	CTGAGATCGGCACGAGGAAC	CTTTCCACAGGGACGAGGTG	Homo sapiens
VEGF A	NM_001171623.1	GCCTTGCTGCTCTACCTCCA	TGTCCACCAGGGTCTCGATT	Homo sapiens
VEGF B	NM_003377.5	CTGGCAGGTAGCGCGAGTAT	GCCACCAGAGGAAAGTGGTG	Homo sapiens
MEOX-1	NM_004527.4	ACTCGGCTCCGCAGATATGA	TCCCTTCACACGCTTCCACT	Homo sapiens
BDNF	NM_170735.5	GGGACTCTGGAGAGCGTGAA	ATTGGGCCGAACTTTCTGGT	Homo sapiens
IGF-1	NM_001111283.2	GCTGAGCTGGTGGATGCTCT	TCATCCACGATGCCTGTCTG	Homo sapiens
IGF-2	NM_000612.5	CAAGTCCGAGAGGGACGTGT	TGGACTGCTTCCAGGTGTCA	Homo sapiens
NGF	NM_002506.3	AGGAGCAAGCGGTCATCATC	ATGTCTGTGGCGGTGGTCTT	Homo sapiens
EPOR	NM_000121.3	CCTGCTTCCCTGGAAGTCCT	CAACCATTTGTCCAGCACCA	Homo sapiens
CD131	NM_000395.2	CCCAGGTCACCAAGGAACAA	GCAATAGTCGCCCACTTGCT	Homo sapiens
EDRF	AY072612.1	TCAATGATCCTCTCGCCTCT	CAGAGCCTTGTCTCGCTCTT	Homo sapiens
CGRP	X02330.1	CCCAGAAGAGAGCCTGTGAC	AAGGCTTTGGAACCCACATT	Homo sapiens
Beta-actin	NM_031144.3	CTCCCTCATGCCATCCTGCG	TCCCTCTCAGCTGTGGTGGT	Rattus norvegicus

GRIA1	NM_031608.1	GGCGTCTGGTGGTTCTTCAC	GCAATTTCCGTCTGCTTTGC	Rattus norvegicus
GRIA2	NM_017261.2	GAGCCAAGGACTCGGGAAGT	ACCAGCATTGCCAAACCAAG	Rattus norvegicus
GRIN3b	NM_133308.2	CTCTTACTCCTCCGCGCTCA	AAAGCACCAGCAGGCAGAAG	Rattus norvegicus
GRIN2A	NM_012573.3	AAGACGGGCTTCCCAACAAT	GGCAGCTTCTGCAATGTGTG	Rattus norvegicus
GRIN2B	NM_012574.1	GGCAGCATTCCTACGACACC	AGCTCTCACCAGCTGGCATC	Rattus norvegicus
MEOX-2	NM_001108837.1	CAGTCTTCTCTGGCCCTCCA	GCAAACATGCCCTCCTCATT	Rattus norvegicus
HIF 1A	NM_024359.2	TCTCGGCGAAGCAAAGAGTC	TGGTGAGCCTCATAACAGAAGC	Rattus norvegicus
BDNF	XM_006234684.4	CCCATGGGTTACACGAAGGA	TATGAACCGCCAGCCAATTC	Rattus norvegicus
NGF	NM_001277055.1	TGTCCCTGAAGCCCACTGGA	AGTGATGTTGCGGGTCTGCC	Rattus norvegicus
CD131	NM_133555.1	TCCGCTTTGGCTGCATCTAT	CCAGGAGGCCAGAGTCCTTT	Rattus norvegicus
EPOR	NM_017002.2	GCCTCCGGATCTCCTCGCTA	GTGGCTGCCCTCTTCTGCTC	Rattus norvegicus
CGRP	NM_017338.2	GCTGGGCACGTACACACAAG	GGGTGGTGGTTTGTCTCCAA	Rattus norvegicus
NOS-1	NM_052799.1	TCCAGATGCCGGCTACACTT	CGTCTCGGGTGTGGTAGGAG	Rattus norvegicus
NOS-3	NM_021838.2	AGTCCAGCGAACAGCAGGAG	GTGCCACGGATGGAAATTGT	Rattus norvegicus
