# Supplementary figures and images for: Carbamoylated Erythropoietin-Induced Cerebral Blood Perfusion and Vascular Gene Regulation
Source: Int J Mol Sci. 2023 Jul 15;24(14):11507. doi: 10.3390/ijms241411507 (PMC10380798; doi:10.3390/ijms241411507)

Figure S2: Experimental design

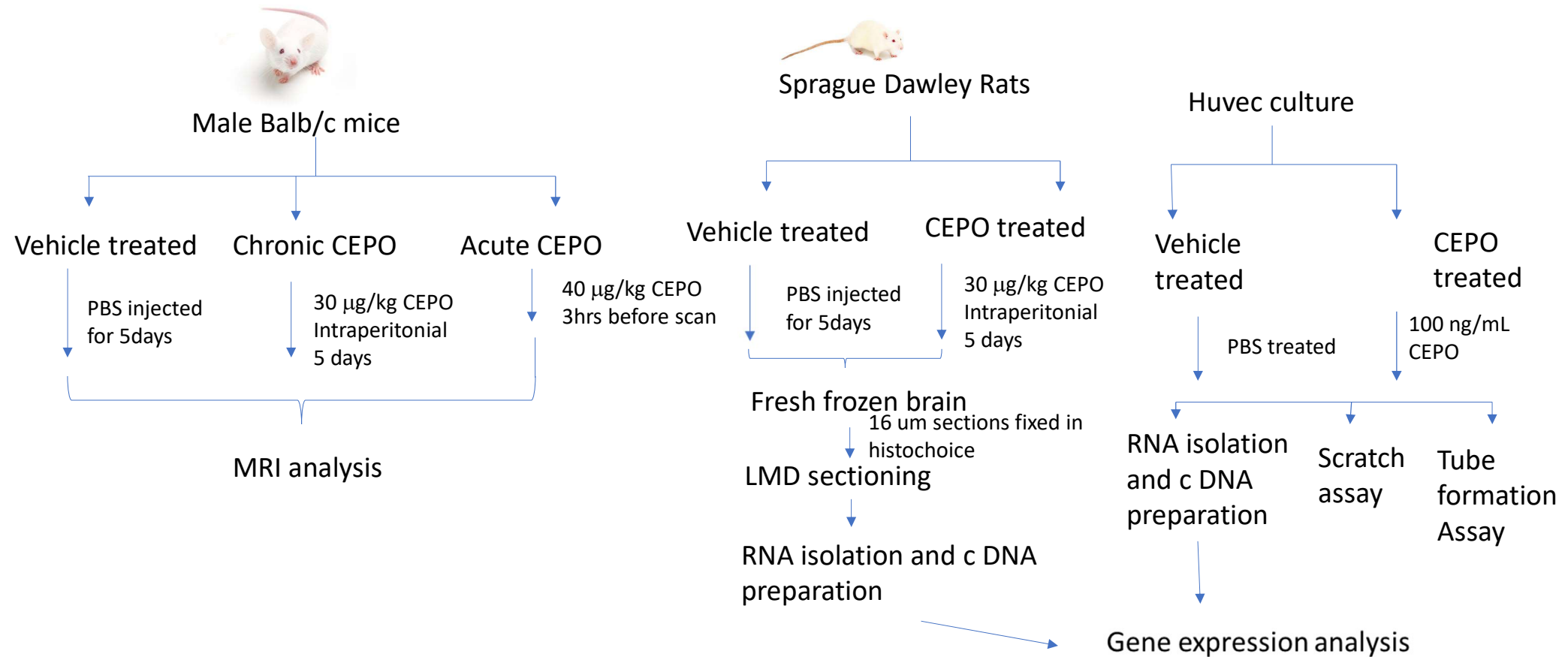

Supplement: Supplementary file 1 [file ijms-24-11507-s001.zip › supplimentary figure s2.pdf]
